# Transcriptome analysis of the parasite *Encephalitozoon cuniculi*: an in-depth examination of pre-mRNA splicing in a reduced eukaryote

**DOI:** 10.1186/1471-2164-14-207

**Published:** 2013-03-28

**Authors:** Cameron J Grisdale, Lisa C Bowers, Elizabeth S Didier, Naomi M Fast

**Affiliations:** 1 Biodiversity Research Centre and Department of Botany, University of British Columbia, Vancouver, British Columbia, Canada; 2 Division of Microbiology, Tulane National Primate Research Center, Covington, LA 70433, USA; 3 Department of Tropical Medicine, Tulane University School of Public Health and Tropical Medicine, New Orleans, LA 70112, USA

## Abstract

**Background:**

The microsporidian *Encephalitozoon cuniculi* possesses one of the most reduced and compacted eukaryotic genomes. Reduction in this intracellular parasite has affected major cellular machinery, including the loss of over fifty core spliceosomal components compared to *S. cerevisiae*. To identify expression changes throughout the parasite’s life cycle and also to assess splicing in the context of this reduced system, we examined the transcriptome of *E. cuniculi* using Illumina RNA-seq.

**Results:**

We observed that nearly all genes are expressed at three post-infection time-points examined. A large fraction of genes are differentially expressed between the first and second (37.7%) and first and third (43.8%) time-points, while only four genes are differentially expressed between the latter two. Levels of intron splicing are very low, with 81% of junctions spliced at levels below 50%. This is dramatically lower than splicing levels found in two other fungal species examined. We also describe the first case of alternative splicing in a microsporidian, an unexpected complexity given the reduction in spliceosomal components.

**Conclusions:**

Low levels of splicing observed are likely the result of an inefficient spliceosome; however, at least in one case, splicing appears to be playing a functional role. Although several RNA decay genes are encoded in *E. cuniculi*, the lack of a few key players could be reducing decay levels and therefore increasing the proportion of unspliced transcripts. Significant proportions of genes are differentially expressed in the first forty-eight hours but not after, indicative of genetic changes that precede the intracellular to infective stage transition.

## Background

Microsporidia possess among the smallest, most compact eukaryotic genomes known [1]. All microsporidia are intracellular parasites and alternate between a thick-walled, extracellular stage (spore) and intracellular stages (meronts, sporonts, and sporoblasts). When triggered, a specialized structure called the polar tube shoots out of the spore and, upon contacting a host cell, creates a passageway into the host [2]. If a host cell is infected, meronts will proliferate, then undergo sporogony before being released from the host cell. The mammalian parasite *Encephalitozoon cuniculi* typically infects humans with compromised immunity due to HIV-infection or immune-suppressive therapy [3,4]. *E. cuniculi* was the first microsporidian to have its genome completely sequenced, and at 2.9 Mb this highly reduced genome possesses many unusual features. It has a reduced coding capacity, encoding less than two thousand protein-coding genes, most of which are shorter than their homologs in yeast [3]. It lacks genes for several biosynthetic pathways and components of the energy-producing tricarboxylic acid cycle. This stripped down genome provides an opportunity to study cellular processes that generally require large, complex sets of components, yet in microsporidia such complexity is reduced, while retaining function. The spliceosome is a large macromolecular machine that is responsible for removing nuclear spliceosomal introns from pre-mRNA via two transesterification reactions [5,6]. In humans, this complex rivals the size of the bacterial ribosome and contains hundreds of protein components and five small nuclear RNAs (snRNAs). Conversely, *E. cuniculi* is only predicted to possess 30 spliceosomal proteins [3]. Such reduced eukaryotes could hold important information about intron and spliceosome evolution as they harbor so few spliceosomal introns (less than 40), and some microsporidia are completely devoid of introns and splicing machinery [7,8].

In a previous study we assessed differences in *E. cuniculi* transcription and spliced transcript levels between intracellular and extracellular life stages [9]. We found that transcripts have much longer untranslated regions (UTRs) and more transcription start sites in the spore stage compared to the intracellular stage. Splicing appears to take place exclusively in the intracellular stage leaving long, unspliced transcripts in the spore, that may play a structural rather than an informational role [9]. Although pre-mRNA splicing occurs, we found no evidence for alternative splicing or mis-splicing [10]. We also found that *E. cuniculi* intron-containing genes have exclusively short 5'UTRs and that, on average, intracellular stage 5'UTR lengths are among the shortest known [11]. Another unusual feature of microsporidian transcription is the presence of overlapping transcripts in the extracellular stage. *E. cuniculi* and the distantly related microsporidian *Antonospora locustae* were both found to have overlapping transcripts [12,13]. However, transcripts in the former often initiate in upstream genes, while those in the latter often terminate in downstream genes [12,13]. These peculiarities of microsporidian molecular biology and the differences in transcripts between extracellular and intracellular life stages led us to conduct a comprehensive investigation of the parasite’s transcriptome during intracellular stages.

Using Illumina HiSeq technology we performed deep RNA sequencing of the *E. cuniculi* transcriptome 24 hr, 48 hr, and 72 hr post-infection. This allowed us to assess spliced transcript and gene expression levels at multiple time points, find novel transcribed regions (NTRs), and improve gene annotations. RNA-seq is an ideal method for examining transcriptomes, as it is relatively unbiased, has greater sensitivity than hybridization methods such as microarrays, and produces high coverage of transcripts [14-17]. We analyzed splicing at all 37 splice junctions to assess the role of these few remaining *E. cuniculi* introns, determined gene expression levels of all annotated genes, found several novel ORFs and, in general, increased our understanding of the dynamic transcriptomes of these unusual parasites.

## Results and Discussion

Genomic analyses of microsporidian species have revealed a number of unusual features that are distinct from other eukaryotes. To date, the microsporidia examined have either done away with introns and splicing machinery entirely, or retain very few of each. How these introns are spliced with greatly reduced machinery, and why so few are retained are questions that pertain both specifically to the evolution of these parasites and, more generally, to intron splicing in eukaryotes. In this study, we present the first transcriptomic analysis of *E. cuniculi*. Intracellular stage genotype 2 *E. cuniculi* was examined at three time-points: 24 hr, 48 hr, and 72 hr after infection of RK13 cells (rabbit kidney fibroblast cell line). A total of 525.9 million reads were produced (Table 1), 40.6 million (7.7%) of which aligned to the *E. cuniculi* genotype 2 reference genome (GenBank accession AEWQ01000000) [18], and 273.5 million (52.0%) of which aligned to the rabbit host (*Oryctolagus cuniculus* reference genome: GenBank accession AAGW02000000). We saw no evidence of cross mapping between host and parasite genomes, as expected, owing to the availability of reference genomes for both organisms and the high level of divergence between them (data not shown). The number of reads mapping to *E. cuniculi* at 24 hr, 48 hr, and 72 hr post-infections were 13.9 million, 17.5 million, and 9.3 million, respectively. This was sufficient coverage to assess splicing and examine gene expression levels at each time-point in order to address questions regarding intron function and evolution, as well as the expression of pathogenesis-related and microsporidia-specific genes.

**Table 1:** Number of reads mapped to parasite and host genomes

**Time-point**	***Encephalitozoon cuniculi***	***Oryctolagus cuniculus***	**Total reads mapped**
T1	13895384	92511829	106407213
T2	17454072	84792245	102246317
T3	9286586	96176009	105462595
Total	40636042	273480083	314116125

The number of reads mapping to *Encephalitozoon cuniculi* and *Oryctolagus cuniculus* at three post-infection time-points are shown.

### Identification of novel transcribed regions

We annotated eleven previously unidentified, transcribed ORFs, three of which have the potential to play a role in pathogenesis. These eleven ORFs are distributed over eight chromosomes. *E. cuniculi* chromosomes were annotated using GLIMMER to find putative ORFs with a minimum length cut-off of either 300 or 150nt [3]. ORFs were used for BLAST searches followed by protein domain identification. This type of annotation leaves open the possibility of ORFs not being annotated due to their small size or lack of known, conserved domains. In order to find novel ORFs that may have been overlooked by the automated annotation software, we examined each chromosome visually using Integrated Genomics Viewer [19] (see Methods for details).

The novel ORF on chromosome 3 (*ECU03_0255*) is a potential candidate for a pathogenesis-related gene involved in cell entry. Although no clear function for this ORF could be predicted from similarity searches, weak (30%) similarity to a viral entry protein could suggest that the product of this ORF functions in host invasion. We discovered two additional ORFs that are so far unique to microsporidia, and therefore may play a role relating to their parasitic lifestyle. Novel ORF *ECU03_0715* has a clear homolog in *E. hellem*, sharing 72% identity over all 116 amino acids. Although not present in all known microsporidian genomes, this ORF shares similarity with genes of unknown function in *Antonospora locustae* and *Nematocida parisii*, two distantly related microsporidia. A second ORF that appears to be microsporidia-specific is *ECU06_0735*, which shares 41% identity over 133 of its 146 amino acids with homeobox domain-containing transcription factors in other *Encephalitozoon* species. The products of these ORFs will require functional analysis to ascertain the cellular roles of their microsporidia-specific protein products.

An additional ORF (*ECU08_1555*) we discovered has no predicted connection to pathogenesis, but may play an important cellular role as it shows similarity to the nucleolar protein NOP10. NOP10 is associated with snoRNAs in ribonucleoprotein complexes that are involved in 18S rRNA production, rRNA pseudouridylation, and are components of the telomerase complex [20]. Additional novel ORFs had very weak similarity to known proteins, and were identified based on transcription signal alone (data not shown). Also, several predicted intergenic regions were transcribed with distinct boundaries but no ORF could be assigned on either strand. These may represent important non-coding RNAs or possibly even unknown selfish genetic elements.

### All coding genes are transcribed in intracellular *E. cuniculi*

The expression data revealed that nearly all 1981 genes had detectable levels of expression in all three time-points (see Additional file 1): all 1981 genes were expressed 24 hr post-infection, 1980 genes were expressed 48 hr post-infection, and 1979 genes were expressed 72 hr post-infection. The twenty genes with highest average expression, in descending order, include spore wall protein 1, RNA-binding domain-containing protein (discussed below), translation elongation factor 1 alpha, actin, histones H2B/H3/H2A/H4, heat shock protein 70, and ribosomal protein L9. The remaining ten genes encode hypothetical proteins with unknown functions. As expected, many highly-expressed genes have housekeeping functions; however, the most highly expressed gene, excluding hypotheticals, is a spore wall protein-encoding gene. This highlights the priority of preparing to form the infective stage, even as early as the first 72 hrs following infection. In summary, essentially all *E. cuniculi* protein-coding genes are expressed during the first three days post-infection in tissue culture.

### High frequency of differentially expressed genes in the first 48 hrs

**Figure 1:**
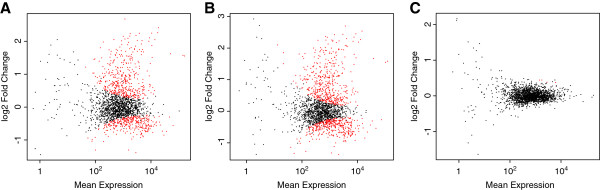
**Differential expression across three post-infection time-points.** Plot of log2 fold change versus mean expression level for all *E. cuniculi* genes. Red dots indicate those genes that are differentially expressed and black dots indicate those that are not. (**A**) Differential expression between 24 hr and 48 hr, (**B**) 24 hr and 72 hr, and (**C**) 48 hr and 72 hr post-infection.

Although nearly all genes are expressed at all time-points, we found an abundance of genes with considerable differences in expression levels between time-points. There were 746 (37.7%) genes differentially expressed between 24 hr and 48 hr post-infection and 867 (43.8%) genes differentially expressed between 24 hr and 72 hr (Figure 1A,B). However, between 48 hr and 72 hr there were only 4 genes differentially expressed (Figure 1C), all with fairly weak fold changes of less than 0.5. This pattern, where many genes are differentially expressed within the first 48 hrs but not after, has implications for the life-cycle of this parasite, such as the possibility that spore formation begins by 48 hr post-infection.

Evidence from expression data suggests that *E. cuniculi* meronts undergo a shift towards producing spore-related genes by 48 hr post-infection. The ten genes with largest positive and negative fold change between 24 hr and the two subsequent time-points includes mostly housekeeping genes and genes encoding hypothetical proteins. An exception to this is polar tube protein 2, whose gene had some of the strongest positive fold changes, both from 24 hr to 48 hr (2.25) and from 24 hr to 72 hr (2.45). Also, the gene encoding polar tube protein 1 showed a similar pattern, with a fold change of 2.05, while the spore wall protein-encoding gene had a fold change of 1.55. This suggests that expression of spore-related genes increases by 48 hr and spore formation could be taking place, however we did not see evidence of spore-specific transcripts with extended 5^′^UTRs [11], even by 72 hr post-infection. This is in line with previous experiments, which have found a spore related gene to have increased expression between 24 hr and 72 hr post-infection [21], and evidence of spore-containing vacuoles beginning at 120 hr post-infection [22].

Housekeeping genes are down-regulated after 24 hr, providing evidence that proliferation is taking place very soon after spore germination and likely for a very brief time. Among the ten most strongly down-regulated genes are several ribosomal protein genes, ubiquitin, an RNA polymerase, two novel ORFs, and several hypothetical protein encoding genes. Down-regulation of housekeeping genes after the 24 hr time-point likely occurs because their expression is high upon germination. We also found that, while many ribosomal protein genes have relatively weak fold changes, they are all negative, further evidence that housekeeping genes as a whole are being down-regulated after 24 hrs. In summary, it seems that spore-specific proteins are produced early in the intracellular life-stage, although spores are likely not formed until after 72 hr post-infection, and housekeeping genes are being down-regulated after 24 hr, possibly as a result of slowing intracellular stage replication rates.

### Analysis of pre-mRNA splicing

#### E. cuniculi has a reduced spliceosome

Gene annotation in *E. cuniculi* identified just 30 ORFs with similarity to spliceosomal components [3], predicting one of the smallest functional spliceosomes known. Several components that are required for viability in yeast are absent in *E. cuniculi*, raising questions about the necessity of these components, the redundancy built into this pathway, and the flexibility of the spliceosome. Also, one of the five RNA components, the U1 snRNA, has not been identified [23]. This suggests that splicing may be occurring without a complete U1 complex, which is involved in the key first step of splicing when the intron is recognized and bound at the 5' splice site [5]. The reduction in *E. cuniculi* spliceosome machinery is severe and is likely to have an effect on the splicing reaction, potentially reducing splicing efficiency.

#### Discovery of introns and splice isoforms

The original genome annotation of *E. cuniculi* predicted 16 introns, almost all of which were in ribosomal protein genes [3]. The number of introns was increased to 34 after a thorough search was performed with a combination of visual and string-search algorithm methods [10]. Many of these new introns were found in non-ribosomal protein-coding genes, which has implications for our understanding of intron retention and evolution in Microsporidia (discussed in [10]). Ranging in size from 22–76 nt, *E. cuniculi* introns are among the smallest spliceosomal introns found in nature, surpassed only by the miniature introns of *Paramecium tetraurelia*[24] and the Chlorarachniophyte nucleomorph genomes [25]. All *E. cuniculi* introns have standard GT-AG boundaries, and relatively strict 5' splice site and branch point motifs (see Additional file 2). This is in line with phylogenetically broad genomic analyses, which have shown that strict splicing motifs are common in intron-poor genomes [26,27]. Utilizing the RNA-seq dataset we confirmed that all previously annotated introns are indeed spliced and are bona fide introns. Also, we found one new intron that creates a novel ORF (*ECU09_1255*), and confirmed splicing of two others that were recently discovered in a comparison of four *Encephalitozoon* species [28]. These three recently detected introns were each confirmed with more than a hundred spliced transcripts, as well as having motifs that are characteristic of *E. cuniculi* introns (Additional file 2).

**Figure 2:**
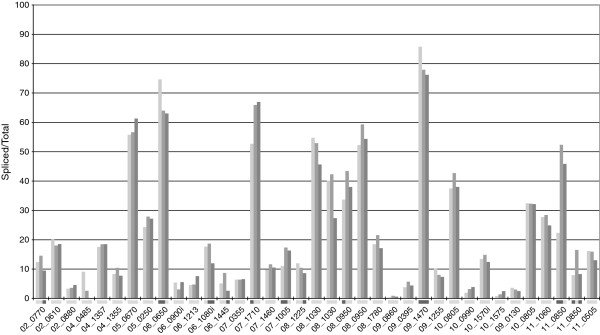
**Splicing levels of all *E. cuniculi *intron-containing genes.** Levels of splicing were determined by measuring the number of spliced and unspliced transcripts and dividing spliced by total transcripts to produce a percentage of splicing. From left to right, splicing levels at 24 hr, 48 hr, and 72 hr are indicated by grey bars. *E. cuniculi* gene names are on the x-axis. Significant differences in splicing levels between time-points are shown by darkened boxes along the x-axis. From left to right, darkened boxes indicate significant differences between 24 hr and 48 hr, 24 hr and 72 hr, and 48 hr and 72 hr.

We have found the first evidence of alternative splicing in a microsporidian parasite. A small proportion of transcripts for three intron-containing genes utilize alternative downstream acceptor sites. Although unexpected to find alternative splicing in such a reduced, streamlined system, the alternative transcripts are so rare that they may represent erroneous splice events. In all cases observed, the alternative isoform represents less than 5% of the reads at the corresponding junction. Despite their low abundance, it is possible that the alternative forms could be utilized as another post-transcriptional regulatory mechanism by inducing rapid decay, as has been hypothesized in *P. falciparum*[29]. If these transcripts were to induce decay this would help explain the rarity at which we observe them. Unfortunately, we lack the tools needed to manipulate decay rates in microsporidia, and therefore cannot test this hypothesis directly. We also see evidence of alternative intron retention, most notably in *ECU11_0850* (Figure 2). In this case the upstream intron is spliced at higher levels than the downstream intron, which would result in some transcripts being truncated at the 3' end, but potentially still functional. Since no genes that function in alternative splicing regulation, such as SR protein family genes, have been found in *E. cuniculi*, we suggest that variation in intron motif features are responsible for differing levels of intron retention within a gene. It has been shown previously that modification to intron motifs can affect splicing efficiency [30]. Therefore, alternative splicing could be playing a minor role in *E. cuniculi* gene expression.

#### Comparative analysis of intron-containing transcripts

We quantified transcript abundance of intron-containing genes to assess levels of intron-retention versus intron removal in order to get a better understanding of the roles of pre-mRNA splicing and RNA decay in *E. cuniculi*. There are several possible scenarios with regards to levels of pre-mRNA splicing and RNA decay. One scenario would be that decay rates are low, and the levels of intron retention or removal are dictated by splicing levels. Another option would be that decay is efficient, creating high levels of spliced transcripts whether or not splicing is efficient, as well. We found that, on average, levels of spliced transcripts in *E. cuniculi* were very low (Figure 2). A staggering 30 of 37 introns (81.1%) had less than 50% of transcripts with introns removed, and 22 (59.5%) of these had below 20% spliced transcripts. Levels of intron-lacking, or spliced, transcripts ranged from less than 5% to over 85%, with one particularly interesting outlier at the high end of the range. The gene *ECU09_1470*, an RNA binding domain-containing protein-coding gene, had previously been noted as unusual for containing the longest *E. cuniculi* intron. In this study we found further reason to examine this gene closely as it had the highest levels of splicing and it was one of the few introns with significant differences in splicing levels between time-points. On the other hand, since all *E. cuniculi* introns contain stop codons or cause frameshifts if not properly removed, it is surprising that the majority of them appear to be spliced at such low levels. For example, over half of the transcripts of thirty of these genes appear to be non-functional because they retain introns. This suggests that decay rates are low (discussed below) and pre-mRNA splicing has a strong influence on levels of transcripts with introns retained or removed.

To assess whether these transcripts were unique to microsporidia or common to parasites and organisms with compact genomes, we performed a similar examination of splicing levels in a free-living and a parasitic fungus. The transcriptomes of *Saccharomyces cerevisiae* and *Candida albicans* encode 306 and 540 introns, respectively [31,32]. The introns of both are similar in size, generally in the 50-1000nt range [33,34]. Although these fungi possess similarly sized spliceosomes that lack over twenty components found in mammals, they encode more than twice as many components as *E. cuniculi*, and therefore, *E. cuniculi* still represents a model of extreme reduction.

Levels of splicing in both *S. cerevisiae* and *C. albicans* were distinctly different from those observed in *E. cuniculi*, with averages of 80% in *S. cerevisiae* and 95% in *C. albicans* (Additional file 3). We found that 32 of 46 (69.6%) *S. cereveisiae* introns were spliced at levels above 80%, while 39 of 46 (84.8%) were spliced at levels above 50%. Splicing levels in *C. albicans* were comparable with 39 of 48 (81.3%) spliced at levels above 80%, and 43 of 48 (89.6%) spliced at levels above 50%. Also, a similar analysis of splicing levels has been performed in the relatively reduced parasitic protist *P. falciparum*, the causative agent of Malaria [29]. The authors found that in this unicellular parasite splicing levels were quite high on average, with a median of five times more spliced reads than intron-retained reads observed [29]. They also note that only 5.6% of introns were spliced at levels below 50% [29]. Therefore, spliced transcript levels in *E. cuniculi* are drastically lower than those in both a fungal and a very distantly related protistan parasite, as well as a free-living fungus. This result, along with the fact that the *E. cuniculi* spliceosome is much more reduced than *P. falciparum* and both fungal species, indicates that it may not be the life-style of the organism that is having such an effect on splicing, but the severe reduction of the spliceosomal machinery. If, over evolutionary timescales, the loss of spliceosomal components resulted in decreases in splicing levels, the reduction of the spliceosome could not have reached its current point unless the levels of intron-containing gene expression were acceptable for cell viability and decay rates increased to compensate for increased intron-containing transcripts. Therefore, the spliceosomal core is likely much smaller than we expect, since mutations in introns and increases in gene expression levels can compensate for decreased splicing levels.

One possible reason for the abundance of transcripts with introns retained is that they could be playing a functional role in gene regulation. For example, several ribosomal protein-coding genes in yeast are known to perform autoregulatory splicing: where the product of the splicing reaction inhibits further splicing by specifically binding to newly made transcripts [35-37]. Other yeast genes have their splicing regulated by environmental stress, such as amino acid starvation [38], or in conjunction with the meiotic cycle [39]. We found 11 of 37 junctions with significant differences in splicing levels between time-points, most with relatively modest changes (Figure 2). Interestingly, one of the few genes with two introns had significant changes in splicing in both introns, including the largest change (30%), and high variability in levels between introns (Figure 2). This provides evidence that splicing may be playing a regulatory role. However, even with nearly a third of intron-containing genes showing differences in splicing levels over the course of infection, nearly all changes are too small to warrant strong evidence of regulatory splicing. Also, we failed to find any strong compensatory role of splicing to moderate expression levels of ribosomal genes, in order to balance their relatively high levels of variability. The low levels of splicing observed do not seem to be the result of regulation at the level of splicing in most cases, however, the splicing patterns of a few genes are indicative of regulation and will require further examination.

Another plausible explanation for the elevated levels of intron-retained transcripts is that RNA decay may not be functioning efficiently in *E. cuniculi*. Since all *E. cuniculi* introns either contain stop codons or induce frameshifts that result in downstream pre-mature stop codons, intron retention should induce transcript degradation by an RNA decay pathway. Metabolic pathways are generally reduced in *E. cuniculi*[3], so complete RNA decay pathways would not be expected. However, *E. cuniculi* appears to have retained a small number of decay proteins, encoding ORFs with similarity to key players including Upf1, Dcp2, Dis3, Dhh1, Ccr4, and Nmd5 (Additional file 4). It is likely that these few decay proteins have evolved to function in the absence of their canonical reaction partners, similar to the spliceosome and DNA repair system [40], as the cell would presumably not be able to function properly without RNA degradation. However, as we predict with spliceosomal functioning, there may be a significant reduction in decay efficiency that could play a part in increasing the proportion of unspliced transcripts present. Yet, to invoke reduced RNA decay as the sole source of these results, decay would have to be very inefficient indeed - a situation that seems unlikely given that no other obvious abnormalities are observed in the transcriptome. Although a formal possibility, it seems unlikely that decay alone is the cause of the high levels of unspliced transcripts. Therefore, the loss of spliceosome components is likely the cause of reduced splicing activity, and in combination with low decay rates, results in a large proportion of unspliced transcripts.

## Conclusions

Assessing the transcriptome of *E. cuniculi* allowed us to improve the genome annotation, uncover novel transcribed regions that could play a role in pathogenesis, discover new introns, and assess levels of intron splicing. We found spliced transcript levels to be surprisingly low on average, most likely as a result of spliceosomal reduction, but with the potential for decreased decay rates to be playing a role. Gene expression levels vary over the course of infection; tremendous numbers of genes are differentially expressed in the first 48 hrs post-infection, suggesting a major genetic change that likely precedes a life-stage change. The reduction of spliceosome and RNA decay pathway components appears to be the cause of decreased splicing efficiency and an accumulation of unspliced, non-functional transcripts. This suggests that a balance is maintained between inefficiency resulting from gene loss, and continued pressure of genome reduction.

## Methods

### RNA preparation

*E. cuniculi* (Genotype II) was cultured in the rabbit kidney fibroblast cell line (CCL-37, American Type Culture Collection, Manassas, VA USA). Intracellular meront stages of *E. cuniculi* appear to bind to the parasitophorous vacuole membrane and thus cannot be physically separated from host cells. Total RNA therefore, was extracted from two biological replicates of RK13 cells in 25cm^2^ tissue culture flasks 24 hr, 48 hr, and 72 hr post-infection using the Ambion RNAqueous kit (Ambion, Austin, TX). Extracted RNA was treated with TURBO DNase (Ambion, Austin, TX) to eliminate any contaminating DNA. RNA quality was assessed on an Agilent Bioanalyzer 2100 (Agilent, Santa Clara, CA) and RNA quantity was measured on a Qubit 2.0 fluorometer (Life Technologies Corp., Carlbad, CA).

### RNA-seq library preparation

A total of six Illumina libraries were prepared according to the TruSeq library preparation protocol (Illumina, Hayward, CA). A total of 4ug of RNA from each of the six DNase-treated samples was used as starting material. Library quality control and pooling were performed by the Biodiversity Research Centre (BRC) sequencing facility (UBC, Vancouver, BC).

### Illumina sequencing and data processing

Paired-end sequencing was performed on an Illumina HiSeq 2000 at the BRC sequencing facility. The six libraries were multiplexed and sequenced in two lanes in order to give two technical replicates for each of the biological replicates, and to help avoid bias associated with a particular flow cell or lane therein [41]. Although paired-end RNA-seq does not account for possible antisense and overlapping transcription, our previous work has indicated that such transcripts are limited to the extracellular spore stage of the parasite [9,11-13].

Raw sequence data was processed and converted to fastq format. Since RNA was obtained from *E. cuniculi* genotype 2 infected RK13 cells, reads were mapped to the genotype 2 reference genome (GenBank accession AEWQ01000000) [18], as opposed to strain GB-M1 [3]. Reads mapping to *E. cuniculi* are available at the NCBI Sequence Read Archive under study accession SRP017112. The short-read aligner Bowtie version 0.12.7 [42] was used for read mapping, using default mismatch parameters, and allowing only a single alignment for each read. Biological and technical replicates showed extremely high levels of correlation (see Additional file 1), as has been seen in previous RNA-seq experiments [31,32,43,44]. SAMtools version 0.1.18 [45] was used to process SAM and BAM alignment files. Alignments were visualized using the Integrative Genomics Viewer version 2.0.7 [19]. Expression levels were measured in the standard fragments per kilobase per million mapped reads (FPKM) format [43]. We found 45 genes with less than twenty reads of coverage in at least one time-point, suggesting that their expression may be the result of background or antisense transcription, and therefore not of biological significance. However, 42 of these encode tRNAs, 5S rRNA, or U2 snRNA, which were not expected to have read coverage following polyA-selected library preparation.

### Assessing splicing efficiency

Attempts were made to use Tophat [46] as a splice junction mapper, however it was not able to detect introns in *E. cuniculi*. Therefore, a custom Bowtie reference was made in order to automate splicing level counts. The sequences of all *E. cuniculi* introns and one hundred flanking nucleotides were obtained from the genome reference [18]. Two reference sequences were created for each intron locus, one containing the intron sequence and one with the intron sequence removed. The flanking sequence was also reduced to 96nt at each end of the splice junction. Therefore, in order for a read to map to one of the reference sequences, it must overlap the splice junction (without the intron) or the intron itself by a minimum of 5nt. The data set was mapped to this reference using Bowtie, producing a SAM output file. SAMtools was used to obtain mapping statistics for the reference sequences, producing counts of the number of reads that map to the spliced and unspliced reference sequences. The number of spliced reads was then divided by the total number of reads covering each splice junction, in order to produce a measure of the splicing levels. Pairwise comparisons of splicing levels for each intron-containing gene were performed with corrected Pearsons’s chi-squared tests in R [47]. Pairs of splicing level values were considered to be significantly different if the chi-squared p-value was less than 0.01. As described above, splicing levels observed are unlikely to result from antisense transcription in this stage of the parasite. Indeed, the presence of significantly different splicing levels across time points for several introns, different splicing levels for two introns in the same gene, and several genes showing high levels of splicing, further supports previous observations that antisense transcription is not widespread in intracellular *E. cuniculi*, and is therefore unlikely to be responsible for the splicing levels observed.

Custom Bowtie reference sequences were prepared for 80 randomly selected introns from *Saccharomyces cerevisiae* and *Candida albicans*, as described above. All RNA-seq reads from the publicly available datasets for *S. cerevisiae* (SRX000559-SRX000564) [31] and *C. albicans* (SRP002852) [32] were mapped against the respective custom reference sequences, allowing spliced and unspliced reads to be counted (as above). Forty-six *S. cerevisiae* and forty-eight *C. albicans* junctions remained after filtering for those with at least 50X coverage.

### Differential gene expression analysis

After mapping with Bowtie, read counts were obtained for all *E. cuniculi* ORFs using HTseq (http://www-huber.embl.de/users/anders/HTSeq/). The read counts were then analyzed for differential expression (DE) using DESeq [48], an R/Bioconductor package [47,49]. A p-value cut-off of 0.01 was used for the DE analysis. The custom *E. cuniculi* gene annotation file used with DESeq was created from the ecotype II genome assembly files [18] using a custom Python [50] script (available upon request).

### Search for novel transcribed regions (NTRs)

The extreme gene-dense nature of the *E. cuniculi* genome made it unreliable to use a custom script to search for NTRs. Therefore, the read alignment files were searched visually for NTRs using IGV. The search parameters used were: a minimum of 10X coverage, no overlap with previously annotated ORFs, and distinguishable borders with regards to the reads mapping to adjacent ORFs, in order to avoid counting untranslated regions.

## Abbreviations

snRNA: Small nuclear RNA; UTR: Untranslated region; ORF: Open reading frame; NTR: Novel transcribed regions; DE: Differential expression; FPKM: Fragments per kilobase of exon per million fragments mapped.

## Competing interests

The authors declare that they have no competing interests.

## Authors’ contributions

CJG extracted RNA, prepared Illumina RNA-seq libraries, performed and interpreted sequence analyses, and drafted the manuscript. LCB and ESD provided parasite material and contributed to interpretation and manuscript preparation, and NMF conceived the study, contributed to the interpretation of the results and drafted the manuscript. All authors read and approved the final manuscript.

## Supplementary Material

Additional file 1 **Gene expression levels.** Expression levels in FPKM are shown for all 1985 *E. cuniculi* genes at three post-infection time-points. Click here for file

Additional file 2 **Intron motifs.** (**A**) Weblogo of 34 *E. cuniculi* intron motifs, showing strict 5' splice site, branch point, and 3' AG. (**B**) Weblogo of three recently discovered introns, with intron motifs that are consistent with currently annotated introns. (**C**) Combined old and new data for a total of 37 introns, showing very little change from (**A**). Click here for file

Additional file 3 **Splicing levels in two fungal species.** Levels of splicing found for 46 *Saccharomyces cerevisiae* introns (**A**) and 48 *Candida albicans* introns (**B**). Splicing level was measured by counting the number of spliced and unspliced transcripts and then dividing spliced by total transcripts to give a percentage of splicing. Click here for file

Additional file 4 **RNA decay genes.** Table of six key RNA decay pathway genes found in *E. cuniculi*. Gene names in yeast and are shown, as well as the protein BLAST e-values. Click here for file
