# Supplementary figures and images for: Transcriptome analysis of the parasite Encephalitozoon cuniculi: an in-depth examination of pre-mRNA splicing in a reduced eukaryote
Source: BMC Genomics. 2013 Mar 28;14:207. doi: 10.1186/1471-2164-14-207 (PMC3629993; doi:10.1186/1471-2164-14-207)

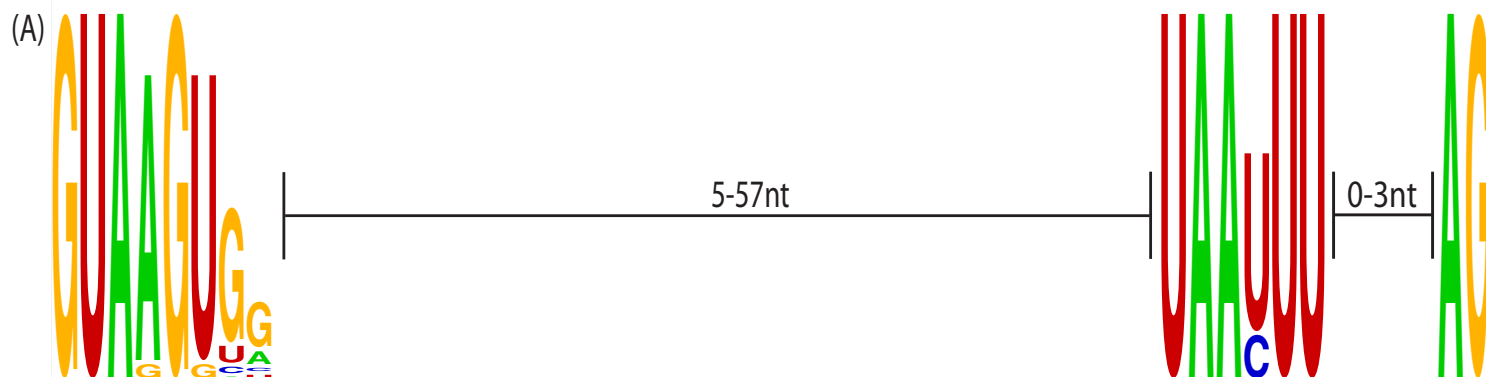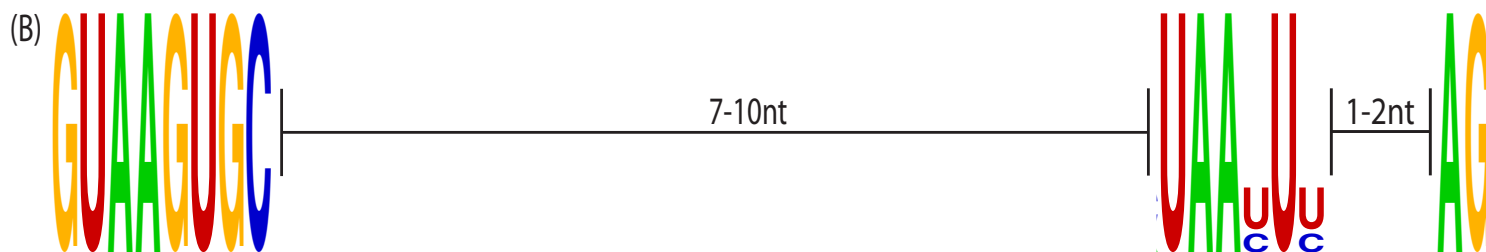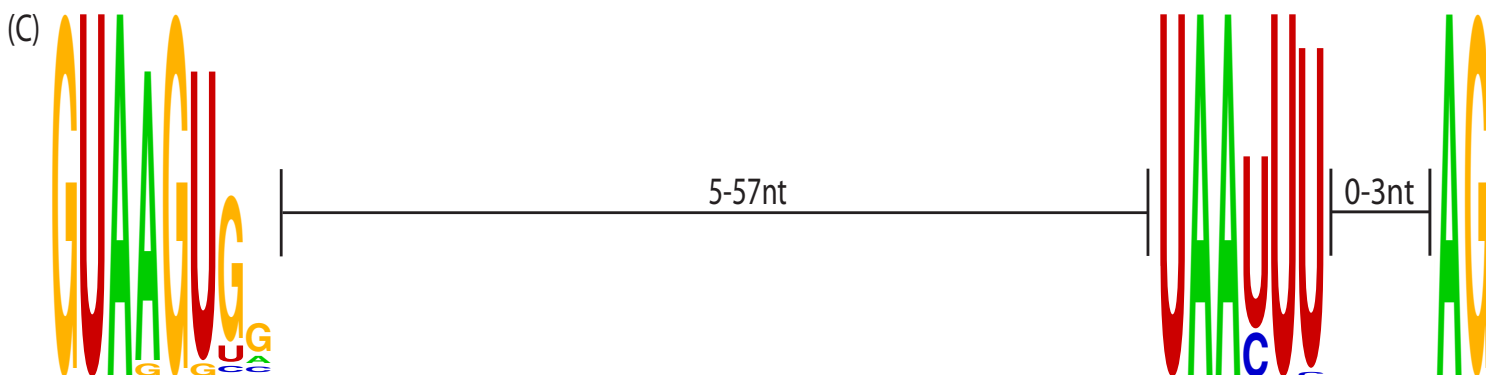

Supplement: Additional file 2 — Intron motifs. (A) Weblogo of 34 E. cuniculi intron motifs, showing strict 5' splice site, branch point, and 3' AG. (B) Weblogo of three recently discovered introns, with intron motifs that are consistent with currently annotated introns. (C) Combined old and new data for a total of 37 introns, showing very little change from (A). [file 1471-2164-14-207-S2.pdf]

(A)

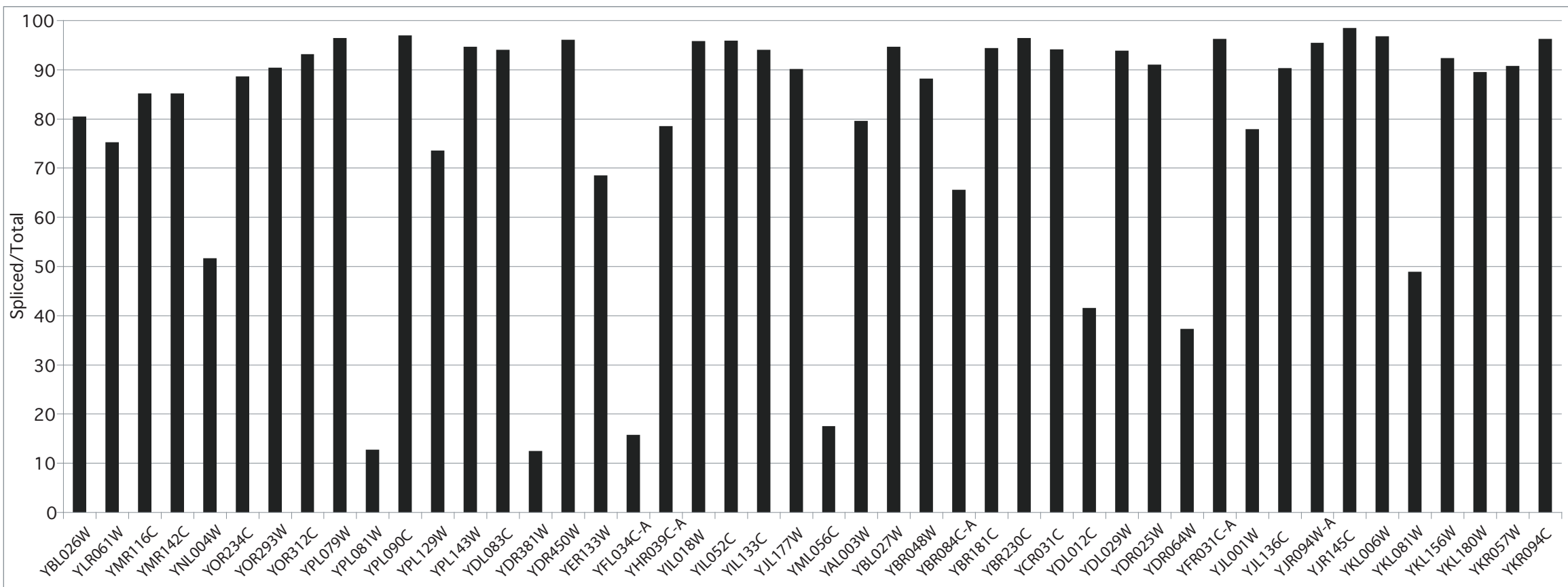

(B)

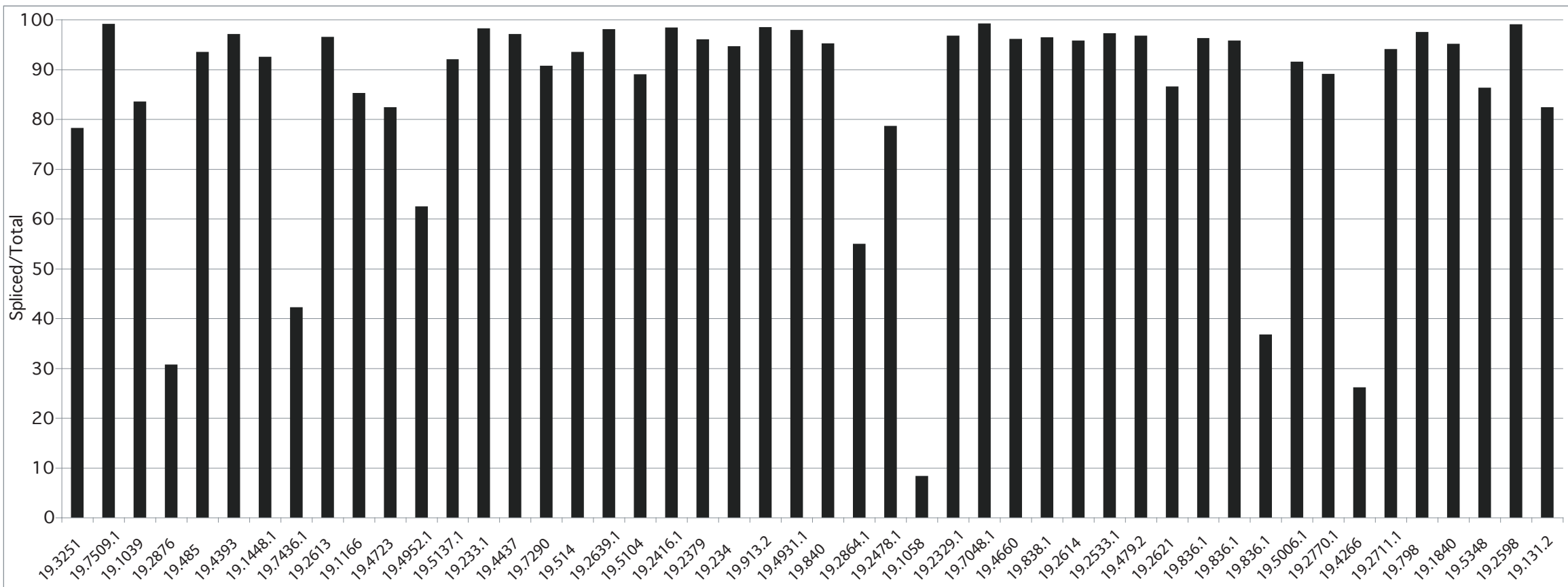

Supplement: Additional file 3 — Splicing levels in two fungal species. Levels of splicing found for 46 Saccharomyces cerevisiae introns (A) and 48 Candida albicans introns (B). Splicing level was measured by counting the number of spliced and unspliced transcripts and then dividing spliced by total transcripts to give a percentage of splicing. [file 1471-2164-14-207-S3.pdf]
